# The Impact of Acute Low-Dose Gamma Irradiation on Biomass Accumulation and Secondary Metabolites Production in *Cotinus coggygria* Scop. and *Fragaria × ananassa* Duch. Red Callus Cultures

**DOI:** 10.3390/metabo13080894

**Published:** 2023-07-28

**Authors:** Alexandra-Gabriela Ciocan, Carmen Maximilian, Elena Monica Mitoi, Radu-Cristian Moldovan, Daniel Neguț, Cristina-Adela Iuga, Florența Elena Helepciuc, Irina Holobiuc, Mihai Radu, Tatiana Vassu Dimov, Gina Cogălniceanu

**Affiliations:** 1 Department of Developmental Biology, Institute of Biology Bucharest of Romanian Academy, 296 Splaiul Independentei Street, 060031 Bucharest, Romania; alexandra.ciocan@ibiol.ro (A.-G.C.); florenta.helepciuc@ibiol.ro (F.E.H.); irisholob@gmail.com (I.H.); gina.cogalniceanu@ibiol.ro (G.C.); 2 Faculty of Biology, University of Bucharest, Splaiul Independentei 91-95, 050095 Bucharest, Romania; tatiana.vassu@bio.unibuc.ro; 3 Department of Proteomics and Metabolomics, Research Center for Advanced Medicine—MedFuture, “Iuliu Hațieganu” University of Medicine and Pharmacy Cluj-Napoca, 4-6 Louis Pasteur Street, 400349 Cluj-Napoca, Romania; moldovan.radu@umfcluj.com (R.-C.M.); iugac@umfcluj.ro (C.-A.I.); 4 IRASM Radiation Processing Department, Horia Hulubei National Institute for R&D in Physics and Nuclear Engineering, Reactorului Street 30, 077125 Magurele, Romania; dnegut@nipne.ro; 5 Department of Pharmaceutical Analysis, Faculty of Pharmacy, “Iuliu Hațieganu” University of Medicine and Pharmacy, Louis Pasteur Street 6, 400349 Cluj-Napoca, Romania; 6 Department of Life and Environmental Physics, Horia Hulubei National Institute for R&D in Physics and Nuclear Engineering, Reactorului Street 30, 077125 Magurele, Romania; mradu@nipne.ro

**Keywords:** *Cotinus coggygria*, *Fragaria* × *ananassa*, callus culture, secondary metabolism, gamma irradiation, UPLC-HRMS, polyphenols, flavonoids, antioxidant activity

## Abstract

*Cotinus coggygria* Scop. (smoketree) and *Fragaria × ananassa* Duch. (strawberry) are two industrially important species due to their composition in bioactive compounds. In this study, we investigated the effects of acute low-dose gamma irradiation (15, 20, 25, 30, 35 and 40 Gy) on two red callus cultures established in smoketree and strawberry. The biomass production, dry weight, content of phenols, flavonoids, monomeric anthocyanins’, index of anthocyanins polymerization and antioxidant activity were evaluated. For the smoketree callus, a negative correlation between irradiation doses and callus biomass accumulation was observed. For the strawberry callus, irradiation did not significantly affect the accumulation of the biomass. An increased dry weight was observed in irradiated smoketree callus, while for treated strawberry callus, a decrease was recorded. Irradiation with 30 Gy was stimulative for polyphenols’ accumulation in both cultures; however, the increase was significant only in the strawberry callus. The flavonoids increased in the 30 Gy strawberry variants, while it significantly decreased in smoketree callus irradiated with 35 and 40 Gy. In irradiated strawberry callus, except for the 25 Gy variant (1.65 ± 0.4 mg C-3-GE/g DW), all treatments caused an increase in anthocyanins’ accumulation. In smoketree, except for the 15 Gy variant (2.14 ± 0.66 mg C-3-GE/g DW), the irradiation determined an increase in anthocyanins synthesis, with the highest value being seen in the 20 Gy variant (2.8 ± 0.94 mg C-3-GE/g DW). According to UPLC-HRMS investigations, an unidentified compound increased by 99% at the 30 Gy dose in strawberry callus, while in smoketree, maslinic acid increased by 51% after irradiation with 40 Gy. The results of this study showed, for the first time, the differential response of two performant callus cultures to low-dose gamma irradiation, a biotechnological method that can be used to stimulate the synthesis of important flavonoids and triterpenes.

## 1. Introduction

Secondary metabolites represent compounds that are produced by plants upon interaction with biotic and abiotic factors in their environment and play important roles in plant adaptation, reproduction and survival [1]. Additionally, these compounds are highly valuable for human health and are used in numerous industries, from food and cosmetics to pharmacology and nutraceuticals [2]. Considering the growing demand by industries for these compounds and the limited capacity of plants to produce them, alternative methods have been developed to ensure the large-scale production of phytochemicals of interest.

In plant biotechnology, in vitro plant cell and tissue cultures play an important role in the production and study of plant secondary metabolites, due to their capacity to produce compounds that are (1) not naturally present in the native plant, (2) found in small quantities or (3) produced in a tissue and season-specific manner [3,4]. In this respect, callus culture is an advantageous and often used technology, which ensures uniform production of compounds all year round, independent from the application of external treatments or plant biological factors [4]. Additionally, various elicitation strategies, which consider the factors present in the plant’s environment can be used in order to modify the cellular metabolism and increase metabolite biosynthesis.

*Cotinus coggygria* Scop. (smoketree) is a shrub species that originated from the temperate area (central and eastern Asia and south-eastern Europe) that has been intensively used in the treatment of cardiovascular [5], oral [6], skin [7,8], digestive [5], respiratory [5] and urinary conditions [5]. The chemical composition of this species has been extensively studied, using all parts of the plant and diverse extraction methods, revealing a complex secondary metabolism, which comprises phenolic acids (gallic acid, chlorogenic acid and rosmaric acid) [9,10,11], flavonoids (sulfuretin, taxifolin, fisetin, myricetin, petunidin-3-glucoside, cyaniding-3-galactoside and delphinidin-3-galactoside) [9,10,11,12,13,14,15,16], hydrolysable tannins (pentagalloyl glucose, galloyl glucose and gallocatechin) [9,17] and terpenes (limonene, myrcene, geranyl acetate, sabinene, terpinolene and α-pinene) [18,19,20,21,22,23,24,25]. Although research using in vitro technologies for micropropagation purposes of different smoketree varieties has been done [26], there are no studies concerning the use of in vitro cultures (hairy roots, suspensions or callus culture) as means for exploiting secondary metabolites of interest.

*Fragaria × ananassa* Duch. (strawberry) is a spontaneous octoploid hybrid between *Fragaria chiloensis* Duch. and *Fragaria virginiana* L., which is highly cultivated for nutritional and pharmacological purposes [27,28]. The strawberry fruit is a rich source of phenolic compounds, mainly anthocyanins, flavonols and flavanols, which possess antioxidant, anti-inflamatory, antimicrobial, antidiabetic, antihypertensive and antineurodegenerative properties [29]. In the last decades, studies have shown that anthocyanin-rich and tannin-rich strawberry fruit extracts that include ellagitannins and proanthocyanidins, also exert anti-cancer effects against multiple human cancer cell types [30,31,32,33].

Considering our previous studies on the secondary metabolites production in *C. coggygria* [34] and *F. ananassa* [35], the acute irradiation with low-dose gamma rays can be an option for secondary metabolism stimulation in smoketree and strawberry red callus cultures. Various studies have tried to explain the influence of diverse doses of gamma rays on morphology, physiology and biochemistry. The findings suggested that gamma rays might exert a hormesis effect, which is a term used to illustrate that the low dose of any agent, applied to a system, shows a positive effect, while its high dose is toxic to the same system [36]. While γ-rays are harmful in high doses, when applied in low doses, this elicitor has been shown to effectively stimulate the biosynthesis of certain secondary metabolites, such as polyphenols, flavonoids and anthocyanins, that in turn could enhance the antioxidant capacity. Low-dose gamma irradiation has been applied with interesting outcomes on in vitro systems, including callus cultures, of several species, such as *Lithospermum erythrorhizon* S. [37], *Rosmarinus officinalis* L. [38], *Panax ginseng* Meyer [39,40], *Nothapodytes foetida* (Slemure) Wight [41], *Stevia rebaudiana* Bert. [42], *Hypericum triquetrifolium* Turra [43], *Artemisia annua* L. [44], *Silybum marianum* L. [45] and *Leontopodium alpinum* Cass. [46].

The aim of the present study was to determine the chemical composition of *Cotinus coggygria* and *Fragaria* × *ananassa* red callus cultures and to investigate the effects of low-dose gamma irradiation on biomass accumulation and main secondary metabolites production.

## 2. Materials and Methods

### 2.1. Plant Material

For callus culture establishment, *C. coggygria* seeds were collected from Șvinița, Mehedinți county, Romania, and *F. ananassa* seeds were obtained from S.C. AGROSEL S.R.L., Câmpia Turzii, Romania (Temptation cultivar, batch 149506) (www.agrosel.ro, accessed on 24 June 2015). In both cases, seedlings were obtained after the seeds were sterilized (0.1% HgCl_2_, 10 min), inoculated on MS medium [47] without hormones and incubated under dark conditions in a growth chamber—Fitotron Weiss-Gallenkamp SCG 120 (Weiss Technik, Loughborough, UK).

For *C. coggygria*, the callus culture was obtained from intact seedlings inoculated on MS induction solid medium [47], supplemented with sucrose, α-naphthylacetic acid (NAA), indole-3-acetic acid (IAA), kinetin (K), 2,4-dichlorophenoxyacetic acid (2,4D) and calcium carbonate [48]. For *F. ananassa*, the callus culture was induced by inoculation of intact seedlings on MS induction solid medium supplemented with sucrose, glucose, 2,4D, NAA, K and vitamin C [49].

Both cultures were placed in the growth chamber at 25 °C under light conditions (photoperiod of 16 h light/8 h dark). *C. coggygria* callus cultures were sub-cultivated on the same fresh medium every 2 weeks, and *F. ananassa* callus cultures were sub-cultivated every 3 weeks.

### 2.2. Irradiation of Callus Cultures Using Gamma Rays

The irradiation experiments were carried out as previously mentioned [46]. Briefly, six acute low-dose gamma rays’ experimental variants were established (15, 20, 25, 30, 35 and 40 Gy). For irradiation, a Gamma Chamber-5000 (Board of Radiation and Isotope Technology, Navi Mumbai, India), with an average transit dose of 4.2 Gy and an average flow of 0.90 Gy/s, was used as ^60^Co-γ source. The experiments were conducted three times and each experimental variant consisted of three repetitions. Each sample was represented by one callus fragment with a mass of 0.3 ± 0.002 g inoculated on a nutritive medium in a Petri dish with a 6 cm diameter. The control variant consisted of non-irradiated samples. After 2 weeks of post-irradiation cultivation in the growth chamber for *C. coggygria,* and 3 weeks for *F. ananassa*, all the experimental variants were used for growth evaluation and biochemical analyses.

### 2.3. Biomass Production Evaluation

Biomass production evaluation was accomplished as previously described [46]. The callus was weighed aseptically on a Highland^®^ HCB302 portable precision balance (Adam Equipment, Kingston, UK). The average growth index (Gi) was calculated using the following formula:

Gi (%) = [(G_1_ − G_0_)/G_1_] × 100,

where G_0_ = weight of callus inoculum before irradiation and G_1_ = callus weight at the end of the cultivation period post-irradiation [43]. To measure the dry weight (DW), 1 g from each fresh callus was dried (80 °C) in an Ecocell oven (MMM Group, Munich, Germany) until a constant weight was achieved. Dry weight was calculated using the following formula:

DW (%) = (G_2_/G_1_) × 100,

where G_1_ = callus weight at the end of the cultivation period post-irradiation and G_2_ = callus weight after dehydration.

### 2.4. Biochemical Analysis

#### 2.4.1. Total Phenolic Content (TPC) Evaluation

Total phenolic content was determined using the Folin–Ciocalteu method [50]. Briefly, 2.5 mL Folin–Ciocalteu (Merck KGaA, Darmstadt, Germany) solution (1:10) and 2 mL 7.5% Na_2_CO_3_ were added to 0.5 mL of diluted extract. The samples were incubated (30 min, room temperature RT) and the absorbance was measured at 765 nm and compared to blank prepared with methanol. Results were expressed as mg of gallic acid equivalents (GAE)/g of DW, based on the gallic acid calibration curve (final concentration: 10–100 µg/mL, R^2^ = 0.9968).

#### 2.4.2. Total Flavonoid Content (TFC) Evaluation

Total flavonoid content was measured using a protocol adapted from Cai et al. (2010) [51]. In brief, 0.5 mL of diluted extract was mixed with 2 mL distilled water and 0.15 mL 5% NaNO_2_. The mixture was incubated (5 min, RT), and then a 10% AlCl_3_ was added. After 6 min at RT, 2 mL of 4% NaOH (*m*/*v*) and 1.2 mL distilled water were added to reach a final volume of 5 mL. The samples were stirred (15 min, RT) and the absorbance for each sample was measured at 510 nm and compared to the blank. Results were expressed as mg of rutin equivalents (RE)/g of DW, based on the rutin calibration curve (final concentration: 100–1000 µg/mL, R^2^ = 0.9996).

#### 2.4.3. Total Monomeric Anthocyanins’ Content (TAC) Evaluation

Total monomeric anthocyanins’ content was determined using the differential pH method [52]. Two dilutions per sample were prepared in 25 mM KCl-HCl buffer pH 1, respectively, in 0.4 M CH_3_COONa- HCl buffer pH 4.5. The absorbance of each sample was measured at 520 nm and 700 nm against a blank prepared with distilled water. The absorbance of each diluted sample was calculated according to the following formula:

A = (A_520_–A_700_)_pH1.0_ − (A_520_–A_700_)_pH4.5_

The difference in pigment absorption at 520 nm and 700 nm is proportional to the pigment concentration:

Monomeric anthocyanin pigment

(mg/l) = (A × MW × DF × 1000)/(ɛ × 1)

where:

MW = molecular weight (449.2 g/mol C-3-G);
DF = dilution factor;
1 = optical path (1 cm);
ε = molar absorptivity (26,900 L × mol^−1^ × cm^−1^ for C-3-G).
Results were expressed as micrograms of cyanidin-3-glucoside equivalents (C-3-GE)/g DW.

#### 2.4.4. Index of Anthocyanins Polymerization (Degradation or Oxidation)

For the index for degradation of anthocyanins, the same method of Giusti and Wrolstad (2001) [52] was used. A volume of 2.8 mL from each diluted sample were mixed with 0.2 mL of K_2_S_2_O_5_ solution or 0.2 mL of distilled water. The difference in absorbance at 420 nm, 520 nm and 700 nm in the bisulphite sample represented the polymeric color. The results were expressed as a percentage of polymerized anthocyanins.

#### 2.4.5. Antioxidant Activity (DPPH) Evaluation

The DPPH (2,2-Diphenyl-1-picrylhydrazyl) assay was performed according to Marxen et al. (2007) [53]. Briefly, 2.25 mL of MeOH and 0.150 mL DPPH (1.27 mM) were mixed with 0.1 mL diluted extract. After incubation (30 min, RT, dark), the absorbance was measured at 515 nm and compared to a reference sample (MeOH). Results were expressed as millimolar of Trolox equivalents (TE)/g of DW, based on the Trolox calibration curve (final concentration: 50–150 µg/mL. R^2^ = 0.9873).

#### 2.4.6. Preparation of Callus Extracts for UPLC-HRMS Analysis

For the UPLC-HRMS characterization of secondary metabolites, three fresh callus samples from each experimental variant were freeze-dried (72 h, −55 °C). Freeze-dried callus (120 mg) was suspended in 6 mL of 70% methanol. Extraction was performed using microwave assisted extraction (MAE) (5 min, 60 °C). After centrifugation (5 min, 8000 rpm), 500 μL of the supernatant were collected and washed 3 times with an equal volume of hexane, for lipid removal. Finally, the diluted extracts (1:40) were used for UPLC-HRMS analysis. Due to the variability of biological samples for all variants (including control), all 3 individual replicates were pooled, and the homogeneous samples were analysed in triplicate.

#### 2.4.7. UPLC-HRMS Analysis

The (+)-catechin reference standard use in this work had a purity of ≥99% and was acquired from Extrasynthese (Genay, France). The organic solvents, methanol and acetonitrile, used for mobile phases and extractions, were of mass spectrometry grade and were purchased from Merck (Darmstadt, Germany). Ultrapure water was produced with the Purelab water filtration system (Elga LabWater, Celle, Germany), while MS grade formic acid was produced by Carlo Erba (Val de Reuil, France).

All analyses were carried out on a Waters I-Class UPLC instrument coupled with a Synapt G2-Si high resolution mass spectrometer (Waters, Milford, MA, SUA). The separations were achieved by reversed phase using a BEH C18 (100 × 2.1 mm) stationary phase (Waters, Milford, MA, USA) and mobile phases formed of 0.1% formic acid (A) and 0.1% formic acid in acetonitrile (B), being pumped at a flow rate of 0.3 mL/min at a temperature of 45 °C. Gradient elution was implemented as follows: 0 min 3%B—10 min 40%B—15 min 98%B. The mass spectrometer was set in resolution mode and negative electrospray ionization by applying 2 kV capillary voltage. Source temperature was 130 °C. Data independent acquisition was achieved in MSE mode between 50 and 1200 *m*/*z* using 2 functions. The collision energies were set to 4 eV (trap) and 2 eV (transfer) for the first function and a ramp between 20–45 eV (trap) for the second function. Leucine-encephalin was used as single point reference (*m*/*z* 554.2615).

#### 2.4.8. Chromatographic Data Processing

Data processing (peak picking, alignment) was carried out using Progenesis QI software (v3.0.79) (Waters, Milford, MA, USA). Compound identification was performed using the Chemspider add-on of the same software and it was carried against two databases (PlantCyc [54] and PhenolExplorer [55]), considering the mass error (max. 10 ppm), fragmentation and isotopic distribution. Major compounds were putatively annotated based on the identification results, but also using available reference standards or based on other literature reports [56,57].

The variation induced by irradiation for each compound was expressed as fold change (FC). This was calculated as the ratio between the compound’s abundance in the irradiated sample and its abundance in the control sample. Thus, a FC of 1.5 equals a 50% increase, while a FC of 0.5 represents a 50% decrease compared to the control sample.

### 2.5. Statistical Analysis

The experimental data obtained were assessed for statistical significance by applying the one-way analysis of variance (ANOVA). The differences between means were determined using the Tukey HSD test, and the Pearson’s correlation test was used to examine the strength of association between variables. All analyses were performed using the software R 3.0.3 (R Foundation for Statistical Computing, Vienna, Austria) [58] and MS Excel. Error bars of graphics represent the standard deviation (±SD) and the level of significance is presented in the footnote of each figure.

## 3. Results

### 3.1. Callus Cultures Characterization

**Figure 1:**
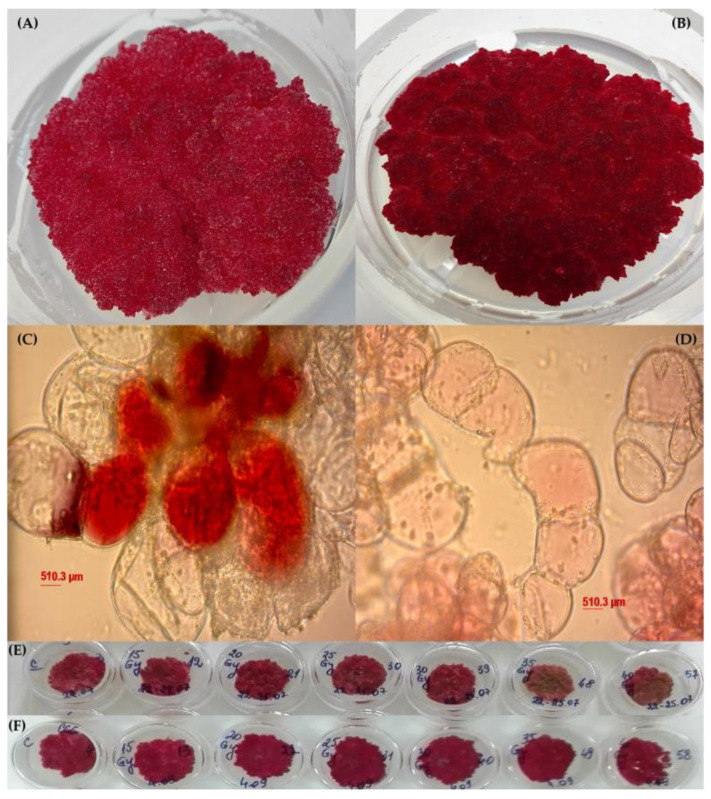
Morphology of untreated (control) *Cotinus coggygria* Scop. and *Fragaria × ananassa* Duch. callus lines: (**A**) macroscopical and (**C**) microscopical aspects of smoketree callus; (**B**) macroscopical and (**D**) microscopical aspects of strawberry callus. Morphology of post-irradiated callus cultures in smoketree (**E**) and strawberry (**F**).

The smoketree and strawberry callus cultures used in this study are macroscopically similar, both displaying a red hyperpigmentation pattern and a relatively compact structure. The strawberry callus presented a more aqueous consistency and neither cultures displayed any morphogenetic centers (Figure 1A,B). On squash micrographs, two different cellular types could be distinguished: some small, round, proliferative cells similar to meristematic-like cells involved in callus growth and other elongated, metabolically active cells, specialized in pigments’ production and accumulation. As previously shown [35], the strawberry callus presented a pigmented stratification structure, with a discontinuous basal layer consisting of grey nuclei, a middle layer in which the small unpigmented cells in division predominate and an upper layer in which the majority are elongated red pigmented cells. In opposition to strawberry, this pigment gradient is not distinguished in the structure of the smoketree callus, its red color being much more homogeneous. However, the squash analyses displayed the presence of clusters of intensely pigmented cells between unpigmented cells in the structure of smoketree callus, while in the strawberry the accumulation of pigments in the cells of the upper layer is much more uniform and not as intense as shown in Figure 1C,D.

Both callus cultures displayed impressive proliferative and metabolic capacities; however, the smoketree callus overproduced secondary metabolites and lost its proliferative capacity after 21 days of subculture. For this reason, the period of subculture was shortened to 14 of days. For the smoketree callus, the average fresh biomass increased 21.8 ± 0.9-fold in comparison to the standardized inoculum (0.3 ± 0.002 g) after 14 days of culture, while the strawberry callus culture registered a 28.1 ± 2.73-fold increase after 21 days of culture. The DW accumulation was 3.66 ± 0.34% in smoketree callus and 4.51 ± 0.19% in strawberry callus. The smoketree culture was more hydrated than the strawberry culture, probably due to the shorter subcultivation period. Total flavonoid and monomeric anthocyanins contents were higher in the smoketree callus (211.33 ± 18.56 mg RE/g DW and 2.24 ± 1.09 µg C-3-GE/g DW, respectively), while total polyphenols content was comparable in two types of red calli. The strawberry callus displayed a higher antioxidant activity (1560.67 ± 239.97 mM TE/g DW), with an almost 2-fold increase over the smoketree callus (784.71 ± 130.25 mM TE/g DW). These results indicate a strong antioxidant activity in the strawberry callus that is not determined quantitatively by the concentration of monitored metabolites, but rather by their chemical composition or the presence of the other classes of compounds.

**Figure 2:**
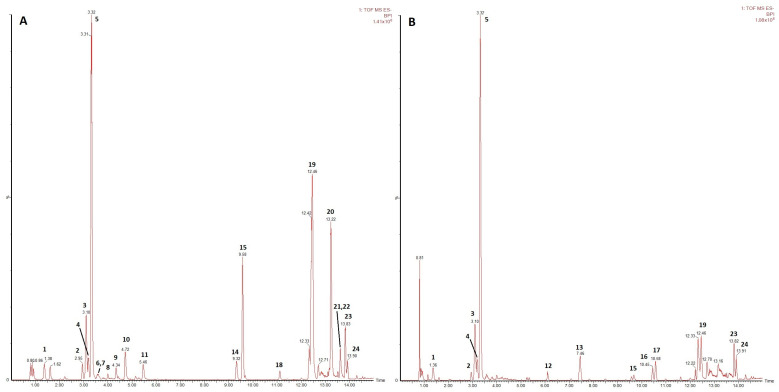
Base peak chromatograms of (**A**) smoketree and (**B**) strawberry methanolic extracts in control (0 Gy) variants. Peak numbers correspond to the compounds from Table 1.

The UPLC-HRMS phytochemical evaluation has shown that the two callus cultures present relatively similar profiles (Figure 2A,B). Both cultures are abundant in condensed tannins and pentacyclic triterpenes, however, in different proportions. In the smoketree callus extract, the condensed tannins represented 34.54%, the most abundant being catechin, and ellagitannins represented 1.75%, while pentacyclic triterpenes constituted approximately 44.40% of the extract, arjunolic and actinic acids being the most abundant compounds of this group (Table 1). The strawberry callus extract contained higher amounts of galloyl glucose and condensed tannins (66.51%), a catechin content of 48.02% and a lower concentration of pentacyclic triterpenes (15.26%) (Table 1).

### 3.2. Impact of Gamma Irradiation on Callus Biomass

**Figure 3:**
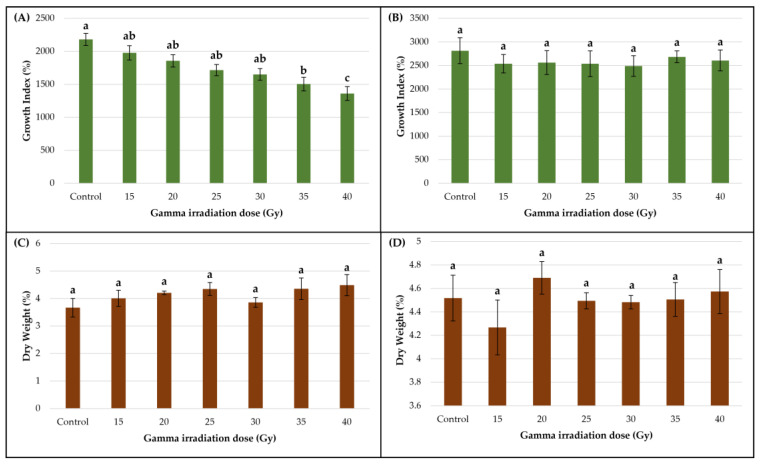
Growth index (Gi) of smoketree (**A**) and strawberry calli (**B**); dry weight (DW) of smoketree (**C**) and strawberry calli (**D**) recorded at the end of the post-irradiation growth period. Results are presented as mean ± SD. Values with different letters are significantly different (*p <* 0.001) One-way ANOVA and multiple pairwise-comparison Tukey tests were used.

Gamma irradiation exerted different effects on the biomass production of the two callus cultures (Figure 3A,B).

For the smoketree callus, a clearly negative correlation between gamma irradiation doses and calli biomass accumulation was observed. The untreated samples displayed the highest growth index (2181.11 ± 90.46%), while significantly lower values were registered in samples treated with the highest doses of 35 Gy (1502.96 ± 102.54%) and 40 Gy (1359 ± 106.12%) (Figure 3A). For the strawberry callus, low-dose gamma irradiation did not significantly affect the biomass accumulation (*p* = 0.06), but recorded values did not exceed the ones measured in the untreated samples (2812.96 ± 276.57%) (Figure 3B). These results suggest that the smoketree callus was more sensitive to irradiation than the strawberry callus, with respect to biomass production. In the 35 Gy and 40 Gy variants, the callus biomass decreased by approximately 31.1% and 37.7%, respectively.

The impact of gamma irradiation on dry weight (DW) was generally not significant; however, an increasing variation tendency was observed in smoketree-treated calli, while for strawberry calli a decreasing tendency was noted. In smoketree callus, samples treated with 40 Gy registered a higher DW (4.49 ± 0.38%) than control (3.66 ± 0.34%), while strawberry samples irradiated with 40 Gy (4.57 ± 0.18%) and 20 Gy (4.69 ± 0.13%) measured higher DW than control (4.51 ± 0.19%).

Regarding the relationship between Gi and DW, Pearson’s correlation test showed that the gamma treatments affected the cultures differently. In smoketree, negative correlations in the 20, 30 and 35 Gy variants were observed, meaning that while Gi decreases, DW increases (Table S1). In strawberry, we observed positive correlations between these parameters in samples treated with 15, 20 and 35 Gy (Table S1).

### 3.3. Impact of Gamma Irradiation on Total Phenolic Content (TPC), Total Flavonoid Content (TFC) and Total Monomeric Anthocyanins’ Content (MAC)

Our results reveal that the irradiation affected the production of total polyphenols, flavonoids and monomeric anthocyanins in different manners.

**Figure 4:**
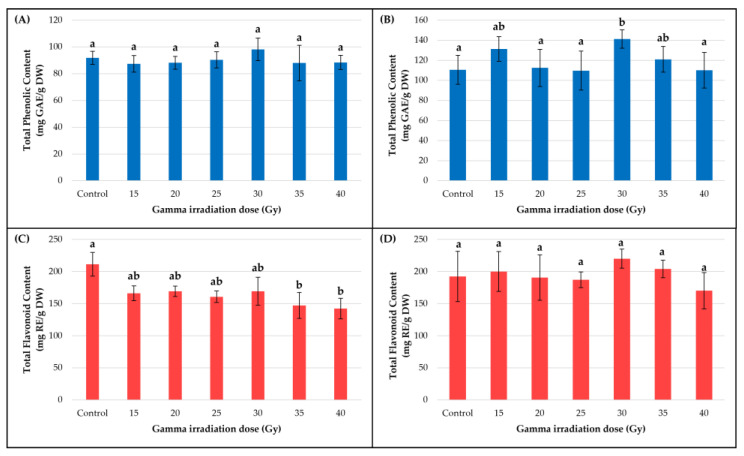
The effect of gamma irradiation on the total phenolic content and total flavonoid content of smoketree (**A**,**C**) and strawberry **(B**,**D**) calli extracts. The data were recorded at the end of the post-irradiation growth period and are presented as mean ± SD. Values with different letters are significantly different (*p <* 0.01). One-way ANOVA and multiple pairwise-comparison Tukey tests were used.

In the smoketree callus culture, TPC varied insignificantly (*p* = 0.48) (Figure 4A); however, a higher polyphenol concentration was measured in the 30 Gy variant (98.21 ± 8.39 mg GAE/g DW) than in the control (91.87 ± 4.88 mg GAE/g DW). With respect to TFC, significantly lower contents (*p* < 0.05) (Figure 3C) were measured in samples treated with 35 Gy (147.21 ± 20.12 mg RE/g DW) and 40 Gy (142.44 ± 15.92 mg RE/g DW) compared to the control (211.33 ± 18.56 mg RE/g DW). The significant decrease in TFC is registered in samples exposed to the highest doses of gamma irradiation and can be associated with a Gi decrease. However, Pearson’s correlation test showed that TFC production was positively correlated with Gi in samples treated with 15 Gy and 40 Gy (Table S1), while a good, positive correlation was remarked between DW and TFC, but only up to the 25 Gy dose (Table S1).

Unlike the smoketree callus, in the strawberry callus, gamma irradiation significantly increased (*p* < 0.01) the TPC, most notably the 30 Gy dose treatment (141.31 ± 9.12 mg GAE/g DW) (Figure 4B). The TFC was not significantly affected by any of the gamma irradiation treatments (*p* = 0.09) (Figure 3D). However, slight increases of TFC were observed in the 15 (200.05 ± 31.06 mg RE/g DW), 30 (220.14 ± 14.75 mg RE/g DW) and 35 Gy variants (204.12 ± 13.49 mg RE/g DW) in comparison to the control (192.29 ± 39.08 mg RE/g DW). According to Pearson’s correlation test, in the strawberry callus, the TPC and TFC were positively correlated in calli irradiated with doses up to 25 Gy and 40 Gy (Table S1). Additionally, there was a significantly positive correlation between Gi and TPC production in calli irradiated with 25 Gy and between DW and TPC in the control (Table S1).

**Figure 5:**
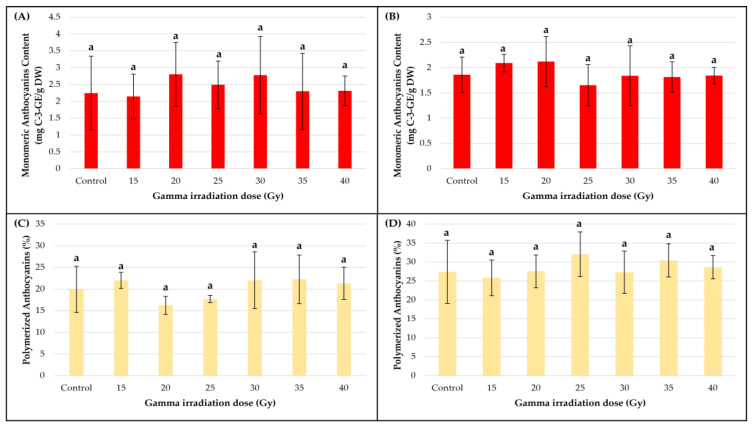
The effect of gamma irradiation on the monomeric anthocyanins content and polymerized anthocyanins of smoketree (**A**,**C**) and strawberry calli (**B**,**D**) extracts. The data were recorded at the end of the post-irradiation growth period and are presented as mean ± SD. Values with same letters are not significantly different.

With respect to MAC production, some of the gamma irradiation treatments had a stimulative effect in both callus cultures, although the increases were not statistically significant (Figure 5A,B). In smoketree, except for the 15 Gy variant (2.14 ± 0.66 mg C-3-GE/g DW), all gamma treatments determined an increase in MAC synthesis in comparison to the control, with the highest value being measured in the 20 Gy variant (2.8 ± 0.94 mg C-3-GE/g DW). In strawberry, except for the 25 Gy variant (1.65 ± 0.4 mg C-3-GE/g DW), all irradiation treatments caused a MAC increase over the control, the highest content being determined in the same 20 Gy variant (2.11 ± 0.49 mg C-3-GE/g DW). In both callus cultures, the index of polymerized anthocyanins did not differ significantly between variants (Figure 5A,B). In smoketree, the decrease of the index, post-irradiation with 20 and 25 Gy, correlated with an increase in MAC, while in strawberry, the samples treated with 25 Gy registered the highest index of polymerization and lowest MAC. Generally, in the smoketree callus, we found negative correlations between MAC production and Gi, except in the 25, 30 and 40 Gy variants (Table S1). In strawberry calli, a good positive correlation was found only in the 20 Gy variant, where the highest MAC was registered (Figure 5B).

The contents of secondary metabolites were correlated to one another in certain experimental variants (Table S1). The smoketree callus culture showed a TFC positively correlated with TPC in samples treated with 25 Gy and 35 Gy, while in strawberry it was shown in samples treated with 15, 20, 25 and 40 Gy (Table S1). The MAC is positively correlated both with TPC and TFC in the 20 Gy and 35 Gy smoketree variants and, respectively, 35 and 40 Gy for strawberry variants (Table S1).

### 3.4. Impact of Gamma Irradiation on Antioxidant Activity (DPPH)

**Figure 6:**
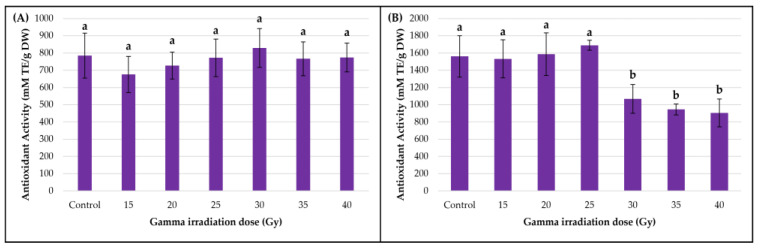
The effect of gamma irradiation on the antioxidant activity of smoketree (**A**) and strawberry (**B**) calli extracts. The data were recorded at the end of the post-irradiation growth period and are presented as mean ± SD. Values with different letters are significantly different (*p <* 0.001). One-way ANOVA and multiple pairwise-comparison Tukey tests were used.

In the smoketree callus culture, the gamma irradiation treatments did not increase the antioxidant capacity measured using DPPH over the control (*p* = 0.52), except for the 30 Gy variant (829.03 ± 111.78 mM TE/g DW) (Figure 6A). In the strawberry callus, slight increases, compared with the control, were observed in the 20 Gy (1586.74 ± 246.23 mM TE/g DW) and 25 Gy variants (1688.74 ± 57.71 mM TE/g DW); however, beginning with the 30 Gy variant (1067.49 ± 166.22 mM TE/g DW), the antioxidant capacity decreased significantly (*p* < 0.001), with the lowest value being registered in samples treated with 40 Gy (905.17 ± 161.56 mM TE/g DW) (Figure 6B). This significant decrease in antioxidant activity post-irradiation with doses higher than 25 Gy could imply that some metabolic modifications occurred that could not be explained by our TPC, TFC or MAC determinations.

### 3.5. UPLC-HRMS Analysis of Secondary Metabolites Post Gamma Irradiation

The irradiation treatments determined the differential production of phenolic compounds in the two callus cultures. Comparative data of the main compounds identified in the smoketree (Table 2) and strawberry callus (Table 3) are expressed as fold change (FC).

In the smoketree callus, the accumulation of four compounds has increased post-irradiation with all treatments and twelve compounds registered a decrease, in comparison to the control, but not in a dose-dependent manner. Here, luteolin-4-glucoside (peak 6) increased by 22% and 26% post-irradiation with 20 and 25 Gy, respectively, while arjunolic acid (peak 19) increased by 9% in the 20 and 35 Gy variants. The accumulation of actinidic (peak 20) and maslinic acids (peak 22) appeared to be stimulated with the increase of gamma dose, the former reaching a 34% increase at 40 Gy and the latter an increase of 51%, also at 40 Gy. Additionally, two procyanidin tetramers (peaks 7, 8) registered increases over control in samples treated with 20, 30, 35 and 40 Gy. On the other hand, the accumulation of hydrolysable (peaks 1, 9–11) and condensed tannins (peaks 2–5) was negatively impacted by all gamma irradiation doses; the most important decrease being seen at the 30 Gy dose, where casuarinin (peak 9) decreased by 61% (Table 2).

In the strawberry callus, one compound (peak 15) was inhibited by all gamma treatments, while two compounds were stimulated by all irradiation doses (peaks 12, 17), with the unidentified compound registering a 99% increase in the 30 Gy variant. This was followed by another unidentified compound (peak 16) that had increased in all variants, except for the 15 Gy, reaching a maximum (71%) in samples treated with 40 Gy. For quercetin-3-glucuronide (peak 12) the maximum increase (35%) was at the 20 Gy dose. Other compounds measured important increases, such as the two pentacyclic triterpenes (peaks 23, 24) that registered a 30% and 47% increase after irradiation with 30 and 40 Gy, respectively. At the same time, irradiation with doses up to 25 Gy inhibited the production of pentacyclic triterpenoid and triterpenes (peaks 19, 23, 24), while the production of condensed tannins (peaks 2–5) was decreased in samples treated with doses up to 30 Gy (Table 3).

## 4. Discussion

The two types of red calli established from *Cotinus coggygria* Scop. and *Fragaria × ananassa* Duch. intact seedlings, used in the elicitation experiments with low doses of gamma radiation, show outstanding biotechnological performances, both in terms of biomass production and the accumulation of secondary metabolites, such as polyphenols, flavonoids or anthocyanins. The procedures for obtaining the two types of calli were the subject of two patent applications applied to OSIM (State Office for Inventions and Trademarks, Bucharest, Romania) [48,49]. UPLC-HRMS characterization showed that the metabolism of the two types of calli is focused towards the production of catechins, pentacyclic triterpenoids, such as arjunolic acid and its isomers and proanthocyanidins, as expected. The two types of calli present macroscopical similarities, but they are structurally distinct. Specifically, in the strawberry callus, a cellular stratification consisting of three layers was distinguished, in relation to the light gradient [35], while the smoketree callus was much more homogeneously pigmented, with cells displaying a very intense red color, a fact demonstrated by the superior synthesis of anthocyanins. Due to its high biosynthetic and accumulation capacity, the subcultivation passage of smoketree callus was shortened from 3 to 2 weeks. Based on our observations, at the end of the 21 days, the growth of callus could have been inhibited by its own synthesized metabolites and not recovered in the subsequent passage.

Our results show that the two callus cultures responded differently to the gamma irradiation treatments, with respect to secondary metabolites’ production and biomass accumulation. The smoketree callus was more sensitive to irradiation than strawberry callus, with the biomass accumulation being significantly affected. We presume that the increasing irradiation doses induced a dehydration or a switch between the ratio of meristematic and specialized cells, favoring the small cells in division and to the detriment of the large, vacuolated cells that accumulate secondary metabolites, hence the increasing DW observed in the smoketree callus (Figure 3A,C). However, the increasing gamma treatments had a generally negative impact on both types of calli. Several studies have reported the potential inhibitory impact of gamma irradiation on callus biomass production in multiple species. *Stevia rebaudiana* (Bert.) Bert calli treated with 5, 10 and 20 Gy registered lower Gi values than control calli [42], while leaf and stem derived *Hypericum triquetrifolium* Turra calli displayed a significantly lower Gi post gamma irradiation with 30 and 40 Gy [43]. In *Gerbera jamesonii* Bolus ex Hooker f., the samples exposed to 20, 30 and 40 Gy presented lower Gi values [60], while in *Leontopodium alpinum* Cass. purple and green calli, the lowest values were registered in the 35 Gy and 40 Gy variants [46].

Although our study concerned an elicitation of secondary metabolism using these experimental systems, slight increases were observed only after applying certain irradiation doses. However, multiple studies have described that gamma irradiation stimulates the synthesis and accumulation of secondary metabolites, such as phenolic and flavonoid compounds, in several species. In *Rosmarinus officinalis* L. callus cultures, irradiation with gradually increasing Gy doses (0, 5, 10, 15, 20 Gy) stimulated the production of TPC and TFC over control, the highest values being measured in the 20 Gy variants (4.38 ± 0.18 mg GAE/g FW and 3.35 ± 0.18 mg QE/g FW) [38]. The same stimulatory effect was reported in *Stevia rebaudiana* (Bert.) Bert. callus cultures, where both TPC and TFC production were increased post gamma irradiation with increasing doses, the highest values being measured in samples treated with 20 Gy (~45 mg GAE/g DW and ~7 mg RE/g DW, respectively) [42]. In our case, a stimulatory effect on TPC and TFC production was recorded post-irradiation with 15, 30 and 35 Gy only in the strawberry callus.

Moghaddam et al. (2011) [61] have shown that both genotype and gamma irradiation treatments can determine differential responses in flavonoid production, in *Centella asiatica* (L.) Urban plantlets. In their study, the CA23 accession treated with 20 Gy and 30 Gy accumulated the highest TFC (16.82 ± 0.02 and 16.83 ± 0.008 mg RE/g DW, respectively), while irradiation with 30 Gy and 40 Gy inhibited the production of flavonoids in the CA03 accession (5.83 ± 0.11 and 5.75 ± 0.03 mg RE/g DW, respectively). The gamma irradiation also produced a differential response in the accumulation of flavonoids in our two types of calli. In the smoketree callus, all administrated doses inhibited the flavonoid production, while in the strawberry callus, certain doses (15, 30 and 35 Gy) slightly stimulated the flavonoids’ accumulation.

In *Ferula gummosa* Bioss. callus cultures, gamma irradiation with 20 Gy (6.41 ± 0.11 mg GAE/g DW) and 25 Gy (6.93 ± 0.11 mg GAE/g DW) determined a significant increase in TPC, in comparison to the control (5.12 ± 0.35 mg GAE/g DW) [62]. In our experiments, higher TPC was measured in both callus cultures treated with 30 Gy, but the 27% increase in polyphenols concentration in strawberry callus was the only significant result.

Overall, the monitored metabolite contents in both cultures did not differ significantly from those measured in untreated samples, proving that the elicitation experiment with the selected doses did not produce the expected results. Some industrially important compounds were stimulated by gamma irradiation in our *C. coggygria* and *F. ananassa* callus cultures. In the smoketree callus, all applied irradiation treatments increased the production of several pentacyclic triterpenoids and triterpenes, tentatively assigned as arjunolic, actinidic and, respectively, maslinic acids and also a flavonoid compound represented by luteolin-4-glucoside. Arjunolic acid is a pentacyclic triterpenoid displaying various bioactivities as anti-fungal, anti-bacterial, anticholinesterase, antitumor and wound healing agents [63], along with protective effects against arsenic-induced oxidative stress at multiple levels [64,65,66,67]. Maslinic acid is a pentacyclic triterpene with antioxidant and cardioprotective effects, and it is capable of reducing the oxidative damage caused by UVB radiation in HFF-1 cells [68] and inhibiting the activation of NF-kB pathways in Sprague-Dawley rats [69]. Actinidic acid was first isolated from unripe kiwi fruit peel [70], and studies have shown that its derivatives exert antidiabetic effects by inhibiting the activity of α-glucosidase [71] and pancreatic lipase [72]. Luteolin-4-glucoside is a flavonoid showing good antioxidant and antiproliferative capacities [73].

Additionally, two procyanidin tetramers accumulation were stimulated at certain doses (20, 30, 35 and 40 Gy) at the expense of the other condensed tannins in the form of dimers or trimers. This superior polymerization of procyanidins as tetramers can also be supported by the increase in the polymerization index of anthocyanins at doses of 30, 35 and 40 Gy. Procyanidins are phenolic compounds that possess antioxidant, antiapoptotic and antiferroptotic effects [74].

We report the presence of some important compounds, for the first time, in *C. coggygria* callus culture: the ellagitannins casuarinin and tellimagrandin II and pentacyclic triterpenes arjunolic, actinidic and maslinic acids. However, further investigations are needed to accurately confirm their identities.

In the strawberry callus, the synthesis of a flavonoid compound, represented by quercetin-3-glucuronide, was stimulated by all irradiation treatments. Quercetin-3-glucoronide is a conjugated flavonoid that exhibits neuroprotective [75], antioxidant, anti-inflammatory and antiapoptotic effects against pulmonary injury [76]. For the pentacyclic triterpenoid and triterpenes determined in this callus, a stimulatory dose was 30 Gy. Research performed in the last two decades revealed that different pentacyclic triterpenoids can possess numerous pharmacological activities, such as anti-cancer, antidiabetic, antioxidant, antiviral, antiprotozoal, cardioprotective, hepatoprotective, anti-bacterial and analgesic [77]. Additionally, pentacyclic triterpenes proved to play an important role in treating vascular disorders caused by hypertension, obesity, diabetes and atherosclerosis [78] and in cancer treatment [79].

## 5. Conclusions

The smoketree and strawberry callus are suitable experimental models for analyzing the content of bioactive compounds, such as polyphenols, antocyanins, flavonoids and evaluation of antioxidant activity, post-treatment with acute low-dose gamma irradiation. A negative correlation between gamma irradiation doses and calli biomass accumulation was observed in smoketree callus. Low-dose gamma irradiation did not significantly affect the biomass accumulation in strawberry callus.

The highest concentration of polyphenols was measured after irradiation with a 30 Gy dose in smoketree and strawberry calli. In the strawberry callus, irradiation with 30 Gy doses induced the maximum concentration of total flavonoids. The monomeric anthocyanins’ values are higher at reduced doses (20 Gy) in irradiated smoketree and strawberry callus cultures. The hormesis effect, with respect to the antioxidant activity, was observed post-low-dose stimulation with 30 Gy in smoketree and 25 Gy in strawberry callus cultures. Characteristic to many biological processes, this effect describes a biphasic response to exposure to increasing amounts of an elicitor (gamma irradiation doses in this study). The UPLC-HRMS analysis revealed the increased synthesis of a pentacyclic triterpene with 51% in smoketree callus, post-irradiation with the 40 Gy dose. In strawberry callus, production of one unidentified compound was stimulated with 99%, at the 30 Gy dose.

In comparison with results from previous studies performed on other species, the low-dose gamma irradiation treatments did not significantly stimulate the biosynthesis of secondary metabolites in our experimental systems, which already displayed impressive phytochemical synthesis capacities. This type of elicitor can be applied to increase the synthesis of certain bioactive compounds.

## 6. Patents

Cogălniceanu, G.C., Mitoi, E.M., Ciocan, A.G., Holobiuc, M.I., Maximilian, R.C., Helepciuc, F.E., Morosanu, A.M. Biotechnological procedure for initiating and obtaining high proliferative cell mass producing bioactive compounds in *Cotinus coggygria* Scop. (Smoketree) and the crude extract, OSIM Romania Patent Application A/00837/17.12.2020 (patent pending).

Cogălniceanu, G.C., Mitoi, E.M., Ciocan, A.G., Holobiuc, M.I., Maximilian, R.C., Helepciuc, F.E., Morosanu, A.M. Biotechnological procedure for initiating and obtaining high proliferative cell mass producing bioactive compounds in *Fragaria x ananassa* Duch. (Strawberry)and the crude extract, OSIM Romania Patent Application A/00808/04.12.2020 (patent pending).

## Figures and Tables

**Table 1:** Major constituents identified in the methanolic smoketree and strawberry calli extracts.

Peak	Rt (min)	*m*/*z* Measured (Da)	*m*/*z* Calculated (Da)	ΔmDa	Formula	Major Fragments	Tentative Identification	Found in		Ident. Level *	Ref.
								*Cotinus*	*Fragaria*		
1	1.37	331.0666	331.0665	0.1	C_13_H_15_O_10_	211.0233, 169.0083, 125.0199	Galloylglucose	Yes	Yes	2	[54,55]
2	2.95	577.1352	577.1346	0.6	C_30_H_25_O_12_	407.0744, 289.0702, 245.0776, 161.0192	Procyanidin dimer	Yes	Yes	2	[54,55,56]
3	3.10	577.1354	577.1346	0.8	C_30_H_25_O_12_	407.0746, 289.0703, 245.0786, 161.0245	Procyanidin dimer	Yes	Yes	2	[54,55,56]
4	3.19	865.1979	865.1979	0.0	C_45_H_37_O_18_	695.1358, 577.1317, 407.0739, 289.0695, 243.0277, 161.0209	Procyanidin trimer	Yes	Yes	2	[54,55,56]
5	3.32	289.0717	289.0712	0.5	C_15_H_13_O_6_	245.0803, 203.0696, 123.0434	(+)-Catechin **	Yes	Yes	1	[54,55]
6	3.59	447.0933	447.0927	0.6	C_21_H_19_O_11_	285.0372	Luteolin-4-glucoside	Yes	No	2	[54,55,57]
7	3.6	1153.2602	1153.2614	−1.2	C_60_H_49_O_24_	865.1873, 575.1165, 447.0907, 407.0699,	Procyanidin tetramer	Yes	No	2	[54,55]
8	4.14	1153.2583	1153.2614	−3.1	C_60_H_49_O_24_	865.1932, 720.1556, 583.1995, 576.1232, 289.0698	Procyanidin tetramer	Yes	No	2	[54,55]
9	4.35	935.0794	935.0790	0.4	C_41_H_27_O_26_	633.0715, 300.9980, 169.0133	Ellagitannin (Casuarinin)	Yes	No	2	[54,55]
10	4.72	937.0946	937.0948	−0.1	C_41_H29O_26_	468.0424, 300.9981, 169.0134	Ellagitannin (Tellimagrandin II)	Yes	No	2	[54,55]
11	5.46	939.1104	939.1103	0.1	C_41_H_31_O_26_	769.0873, 617.0767, 169.0133	Gallotannin (Pentagalloyl glucose)	Yes	No	2	[54,55,56]
12	6.11	477.1033	477.1033	0.0	C_22_H_21_O_12_	314.0402, 300.0200, 285.0390, 271.0211, 243.0244	Quercetin-3-glucuronide	No	Yes	2	[54,55]
13	7.45	711.3965	-	-	-	503.3334	*Unidentified*	No	Yes	5	[54,55]
14	9.32	695.4013	-	-	-	487.3402	*Unidentified*	Yes	No	5	[54,55]
15	9.57	695.4014	-	-	-	487.3407	*Unidentified*	Yes	Yes	5	[54,55]
16	10.45	503.3374	503.3372	0.2	C_30_H_47_O_6_	485.3228, 453.3029	*Unidentified*	No	Yes	4	[54,55]
17	10.58	503.3374	503.3372	0.2	C_30_H_47_O_6_	485.3263, 453.2953, 441.3333, 421.3064, 409.3079	*Unidentified*	No	Yes	4	[54,55]
18	11.11	693.3853	693.3850	0.3	C_37_H_57_O_12_	485.3254	Triterpenoid (Phytolaccoside)	Yes	No	3	[54,55]
19	12.45	487.3430	487.3423	0.7	C_30_H_47_O_5_	469.3312, 423.3254, 407.3299	Pentacyclic triterpenoid (Arjunolic acid)	Yes	Yes	3	[54,55]
20	13.23	485.327	485.3267	−0.3	C_30_H_45_O_5_	467.1826	Pentacyclic triterpenoid (actinidic acid)	Yes	No	3	[54,55]
21	13.54	633.3794	633.3791	0.3	C_39_H_53_O_7_	-	*Unidentified*	Yes	No	4	[54,55]
22	13.62	471.3477	471.3474	0.3	C_30_H_47_O_4_	381.2289	Pentacyclic triterpene (Maslinic acid)	Yes	No	3	[54,55]
23	13.82	471.3477	471.3474	0.3	C_30_H_47_O_4_	-	Pentacyclic triterpene	Yes	Yes	3	[54,55]
24	13.91	471.3477	471.3474	0.3	C_30_H_47_O_4_	-	Pentacyclic triterpene	Yes	Yes	3	[54,55]

* Identification level confidence [59]; ** Identified by comparison with reference standards.

**Table 2:** Impact of gamma irradiation on the major constituents of smoketree calli extracts.

Peak	Compound	Gamma Irradiation Dose					
		15 Gy	20 Gy	25 Gy	30 Gy	35 Gy	40 Gy
1	Galloyl glucose	0.91	0.90	0.83	0.74	0.81	0.76
2	Procyanidin dimer	0.85	0.91	0.74	0.77	0.74	0.67
3	Procyanidin dimer	0.91	0.97	0.88	0.90	0.92	0.89
4	Procyanidin trimer	0.90	0.98	0.86	0.90	0.91	0.87
5	(+)-Catechin	0.90	0.93	0.84	0.84	0.86	0.83
6	Luteolin-4-glucoside	1.10	1.22	1.26	1.12	1.11	1.10
7	Procyanidin tetramer	0.93	1.06	0.95	1.02	1.08	1.08
8	Procyanidin tetramer	0.94	1.14	1.00	1.07	1.08	1.08
9	Ellagitannin (Casuarinin)	0.87	0.64	0.66	0.39	0.52	0.58
10	Ellagitannin (Tellimagrandin II)	0.82	0.76	0.78	0.56	0.65	0.65
11	Gallotannin (Pentagalloyl glucose)	0.82	0.86	0.81	0.70	0.72	0.66
14	Unidentified	0.88	1.05	0.98	0.82	0.76	0.67
15	Unidentified	0.86	0.99	0.86	0.77	0.74	0.64
18	Triterpenoid (Phytolaccoside)	0.91	0.97	0.94	0.80	0.87	0.72
19	Pentacyclic triterpenoid (Arjunolic acid)	1.05	1.09	1.06	1.07	1.09	1.12
20	Pentacyclic triterpenoid (Actinidic acid)	1.05	1.09	1.11	1.16	1.29	1.34
21	Unidentified	0.88	1.07	0.85	1.10	1.09	1.11
22	Pentacyclic triterpene (Maslinic acid)	1.08	1.19	1.20	1.27	1.37	1.51
23	Pentacyclic triterpene	0.97	0.98	0.84	0.82	0.78	0.77
24	Pentacyclic triterpene	0.97	0.93	0.78	0.74	0.65	0.62

Color scheme explanation: yellow—FC = 1.0; red—FC ≤ 0.8; green—FC ≥ 1.2.

**Table 3:** Impact of gamma irradiation on the major constituents of strawberry calli extracts.

Peak	Compound	Gamma Irradiation Dose					
		15 Gy	20 Gy	25 Gy	30 Gy	35 Gy	40 Gy
1	Galloyl glucose	1.03	0.93	0.98	0.91	0.75	0.59
2	Procyanidin dimer	0.96	0.98	0.99	0.96	1.04	1.02
3	Procyanidin dimer	0.94	0.99	1.01	0.98	1.03	1.02
4	Procyanidin trimer	0.93	0.98	0.98	0.94	1.05	1.05
5	Catechin	0.97	1.00	1.01	0.99	1.04	1.03
12	Quercetin-3-glucuronide	1.03	1.35	1.22	1.08	1.13	1.27
13	Unidentified	1.01	1.08	1.07	0.98	0.89	0.94
15	Unidentified	0.85	0.60	0.62	0.75	0.44	0.40
16	Unidentified	0.98	1.24	1.26	1.23	1.37	1.71
17	Unidentified	1.22	1.36	1.64	1.99	1.48	1.61
19	Pentacyclic triterpenoid (Arjunolic acid)	0.98	0.78	0.89	1.12	0.88	0.98
23	Pentacyclic triterpene	1.05	0.77	0.97	1.30	0.99	1.07
24	Pentacyclic triterpene	0.99	0.81	0.89	1.10	1.15	1.47

Color scheme explanation: yellow—FC = 1.0; red—FC ≤ 0.8; green—FC ≥ 1.2.

## Data Availability

All data used and obtained during this study are included in this research paper as Figures, Tables and Supplementary Files.

## Supplementary Materials

The following supporting information can be downloaded at https://www.mdpi.com/article/10.3390/metabo13080894/s1. Table S1: Pearson’s correlation coefficient between analysed parameters: growth index (Gi), dry weight (DW), content of polyphenols (TPC), flavonoids (TFC), monomeric anthocyanins (MAC) and antioxidant activity (DPPH).
