# Supplementary material for: The Impact of Acute Low-Dose Gamma Irradiation on Biomass Accumulation and Secondary Metabolites Production in Cotinus coggygria Scop. and Fragaria × ananassa Duch. Red Callus Cultures
Source: Metabolites. 2023 Jul 28;13(8):894. doi: 10.3390/metabo13080894 (PMC10456250; doi:10.3390/metabo13080894)
Supplement: Supplementary file 1 [file metabolites-13-00894-s001.zip › metabolites-2513723-supplementary.pdf]

Table S1. Pearson's correlation coefficient between analysed parameters: growth index (Gi), dry weight (DW), content of polyphenols (TPC), flavonoids (TFC), monomeric anthocyanins (MAC) and antioxidant activity (DPPH).

**Species**	**Exp. variant**	**Gi-DW**	**Gi-TPC**	**Gi-TFC**	**Gi-MAC**	**Gi-DPPH**	**DW-TPC**	**DW-TFC**	**DW-MAC**	**DW-DPPH**	**TPC-TFC**	**TPC-MAC**	**TPC-DPPH**	**TFC-MAC**	**TFC-DPPH**	**MAC-DPPH**
		r value	r value	r value	r value	r value	r value	r value	r value	r value	r value	r value	r value	r value	r value	r value
C. coggygria	Control	0.373	-0.861	0.161	-0.966	-0.423	0.148	0.975	-0.598	0.682	0.361	0.703	0.824	-0.408	0.825	0.177
	15 Gy	0.755	-0.850	0.913	-0.855	-0.985	-0.297	0.956	-0.985	-0.855	-0.561	0.454	0.748	-0.992	-0.969	0.930
	20 Gy	-0.970	-0.474	-0.949	-0.722	0.834	0.671	**0.997***	0.866	-0.941	0.726	0.951	-0.881	0.902	-0.964	-0.983
	25 Gy	0.224	0.224	-0.069	0.923	0.797	**0.999***	0.958	0.580	-0.409	0.951	0.597	-0.389	0.323	-0.652	0.505
	30 Gy	-0.968	0.788	0.183	0.933	**0.998***	-0.916	-0.420	-0.815	-0.979	0.748	0.515	0.815	0.181	0.227	0.916
	35 Gy	-0.969	0.319	0.198	-0.069	0.666	-0.079	0.046	0.310	-0.465	0.992	0.922	0.919	0.963	0.862	0.697
	40 Gy	0.871	0.333	0.877	0.968	-0.631	0.753	0.530	0.965	-0.169	-0.158	0.556	0.520	0.732	-0.925	-0.420
F. ananassa	Control	0.725	0.732	-0.374	0.971	-0.540	**0.999****	0.365	0.540	-0.971	0.356	0.548	-0.968	-0.585	-0.577	-0.324
	15 Gy	0.959	-0.544	-0.853	0.739	0.746	-0.758	-0.965	0.520	0.529	0.901	0.161	0.151	-0.281	-0.291	**0.999****
	20 Gy	0.858	-0.255	0.177	0.950	0.334	-0.715	-0.352	0.655	0.770	0.906	0.059	-0.996	0.457	-0.867	0.024
	25 Gy	-0.985	**0.998***	0.910	0.534	0.996	-0.993	-0.966	-0.385	-0.968	0.929	0.491	0.991	0.136	0.873	0.601
	30 Gy	-0.508	-0.763	-0.739	0.599	0.102	0.944	-0.202	0.384	-0.908	0.129	0.059	-0.720	-0.981	0.593	-0.735
	35 Gy	0.859	-0.034	-0.558	-0.502	0.555	-0.540	-0.903	-0.873	0.053	0.848	0.881	0.811	**0.997***	0.379	0.439
	40 Gy	0.769	-0.785	-0.668	-0.496	-0.847	-0.209	-0.039	0.171	-0.313	0.985	0.927	0.994	0.977	0.961	0.881

*$p<0.05$; **$p<0.01$; ***$p<0.001$, according to Pearson's correlation coefficient.
